# ADH5/ALDH2 dehydrogenases and DNA polymerase theta protect normal and malignant hematopoietic cells from formaldehyde challenge: therapeutic implications

**DOI:** 10.1038/s41375-025-02687-3

**Published:** 2025-07-10

**Authors:** Jessica Atkins, Anna-Mariya Kukuyan, Monika Toma, Malgorzata Drzewiecka, Umeshkumar Vekariya, Adam Karami, Margaret Nieborowska-Skorska, Reza Nejati, Emir Hadzijusufovic, Peter Valent, Tomasz Stoklosa, Tomasz Sliwinski, Mariusz Wasik, Tomasz Skorski

**Affiliations:** 1 Fels Cancer Institute for Personalized Medicine, Lewis Katz School of Medicine, Temple University, Philadelphia, PA USA; 2 Department of Molecular Genetics, Faculty of Biology and Environmental Protection, University of Lodz, Lodz, Poland; 3 Department of Pathology, Fox Chase Cancer Center, Philadelphia, PA USA; 4 Department of Internal Medicine I, Division of Hematology and Hemostaseology, Medical University of Vienna, Vienna, Austria; 5 Ludwig Boltzmann Institute for Hematology and Oncology, Medical University of Vienna, Vienna, Austria; 6 Department of Tumor Biology and Genetics, Medical University of Warsaw, Warsaw, Poland; 7 Department of Cancer and Cellular Biology, Lewis Katz School of Medicine, Temple University, Philadelphia, PA USA; 8 Nuclear Dynamics and Cancer Program, Fox Chase Cancer Center, Philadelphia, PA USA

**Keywords:** Acute myeloid leukaemia, Myeloproliferative disease

## Abstract

Normal hematopoietic stem and progenitor cells (HSPCs) are exposed to physiological levels of formaldehyde but occasionally may be challenged by high levels of formaldehyde generated by endogenous and exogenous sources. In addition, leukemia cells stressed by oncogenic mutations continuously produce excessive amounts of formaldehyde. Here, we show that DNA polymerase theta (Polθ) cooperates with alcohol dehydrogenase 5 (ADH5) and aldehyde dehydrogenase 2 (ALDH2) to protect healthy and malignant HSPCs challenged by formaldehyde. ADH5 and ALDH2 metabolize formaldehyde while Polθ-mediated DNA repair by microhomology-dependent end-joining (TMEJ) protects cells from the lethal effect of DNA double strand breaks resulting from formaldehyde-mediated DNA-protein crosslinks. Genetic or pharmacological targeting of ADH5 or ALDH2 enhanced the effect of Polθ inhibitors in leukemic cells. Thus, ADH5/ALDH2 cooperate with Polθ to protect normal HSPCs sporadically challenged by high levels of formaldehyde, and inhibition of Polθ and ADH5 or ALDH2 may exert an anti-leukemic effect.

## Introduction

DNA can be damaged by various metabolites including aldehydes such as formaldehyde which may lead to various forms of cancer and/or organ dysfunction [1–3]. Formaldehyde is a highly reactive and carcinogenic aldehyde that can accumulate in cells from both endogenous and exogenous sources [1, 3, 4]. Formaldehyde can be produced within the cell by one-carbon metabolism, methanol metabolism, lipid peroxidation, and DNA demethylation [2–6]. Exogenous sources include various industrial and consumer products, cigarette smoke, fires, and automotive exhaust [2, 4].

Cells are exposed to physiological levels and occasionally to higher concentrations of formaldehyde. The latter can be generated by exogenous sources or endogenously by transcriptional reprogramming associated with the differentiation of hematopoietic cells or stimulation of one-carbon metabolism by oncogenic tyrosine kinases (OTKs) in acute myeloid leukemia (AML) and myeloproliferative neoplasms (MPN) [4, 7–10]. Evidence suggests that exposure to formaldehyde can lead to myeloid leukemia and/or bone marrow failure, which likely results from DNA damage [4, 11, 12].

Formaldehyde leads to the formation of interstrand crosslinks (ICLs), DNA-protein crosslinks (DPCs) and oxidative lesions [2, 3, 13–16]. ICLs are cytotoxic lesions that covalently link opposite strands of DNA whereas DPCs are bulky conjugates that covalently link DNA and neighboring proteins [3, 17]. ICLs and DPCs can impede DNA replication and transcription [3, 17]. If these modifications are not repaired properly, they can stall and destabilize replication forks, resulting in the formation of DNA double strand breaks (DSBs) which can be lethal to cells [13, 14]. For protection from formaldehyde-mediated DNA damage, hematopoietic cells rely on metabolic enzymes and DNA repair systems.

Alcohol dehydrogenase 5 (ADH5/GSNOR) and aldehyde dehydrogenase 2 (ALDH2) are two key enzymes in preventing the accumulation of formaldehyde in hematopoietic cells. ADH5 is the primary defense against formaldehyde while ALDH2 provides a backup [18–20]. ADH5 and ALDH2 metabolize formaldehyde to produce the less toxic metabolite formate which is then utilized in other biological processes [18–20]. While mutations in *ADH5* or *ALDH2* are associated with increased risk of cardiovascular disorders, mutations of the latter have also been associated with certain types of cancer [21–24]. Patients carrying mutations in both *ADH5* and *ALDH2* develop Aldehyde Degradation Deficiency Syndrome which mimics the clinical features of other rare bone marrow failure syndromes like Fanconi anemia [19, 20, 25].

Formaldehyde-mediated DNA lesions are not all equally genotoxic and mutagenic. Therefore, it is crucial to better understand which lesions underpin formaldehyde genotoxicity and to characterize the DNA repair pathways that can repair them. The Fanconi anemia (FA) DNA repair pathway plays a critical role in the repair of formaldehyde-mediated DNA damage (e.g., ICLs) generated under steady-state conditions within HSPCs [26]. It has been postulated that the cellular genome is protected from the toxicity of intracellularly generated formaldehyde by a two-tiered mechanism: ADH5 and ALDH2 are responsible for the clearance of formaldehyde (Tier-1) and the FA DNA repair pathway resolves formaldehyde-induced ICLs (Tier-2) [2, 3, 19]. However, it remained unknown if these two cooperating mechanisms would provide enough protection when cells are challenged with high levels of formaldehyde, generating more extensive DNA damage.

We previously reported that DNA polymerase theta (Polθ) (encoded by *POLQ* gene) plays a critical role in protecting cells from the toxic effect of formaldehyde-induced DPC-associated DSBs [27]. Polθ mediates microhomology-dependent end-joining (TMEJ), restarts stalled replication forks, and fills single-stranded DNA (ssDNA) gaps during replication fork progression [28–32]. Inhibition of Polθ is synthetically lethal in *BRCA1/2*-mutated homologous recombination (HR)-deficient tumors, which is being explored therapeutically [33].

Here, we show that Polθ provides a third-tier of protection in normal and malignant HSPCs challenged by high concentrations of formaldehyde. We also demonstrate that simultaneous targeting of Polθ and ADH5 or ALDH2 may have therapeutic potential in HR-deficient leukemias, and those harboring high levels of endogenous formaldehyde triggered by OTKs.

## Materials and methods

### Mouse husbandry

Wild-type *(wt)* and *Polq*^*−/−*^ mice were obtained from Jackson Laboratories (JAX 000664, JAX 006194). *Adh5*^*−/−*^ mice were provided by Jonathan Stamler (Institute for Transformative Molecular Medicine, Case Western Reserve University School of Medicine, Cleveland, OH) and *Aldh2*^*−/−*^ mice were provided by Lopa Mishra (The Institute for Bioelectronic Medicine, Feinstein Institutes for Medical Research & Cold Spring Harbor Laboratory, Department of Medicine, Division of Gastroenterology and Hepatology, Northwell Health, Manhasset, NY). *Adh5*^*−/−*^ and *Aldh2*^*−/−*^ mice were cross-bred with *Polq*^*−/−*^ mice to generate *Adh5*^*−/−*^*;Polq*^*−/−*^ and *Aldh2*^*−/−*^*;Polq*^*−/−*^ mice (Supplementary Fig. S1A–D and Supplemental Materials and Methods).

### Cell lines and treatment

Nalm6 parental and Nalm6-*RAD54*^*−/−*^ cells were described before [34]. Cells were treated with the indicated inhibitors and/or combinations for 48 h followed by clonogenic test in methylcellulose as described before [34].

### AML and MPN patient cells

AML201 primary sample was previously described [35] and single-cell targeted DNA sequencing (sctDNA-seq) revealed that 93.48% of cells carried FLT3(D593ins) mutation. For AML1, bulk sequencing found FLT3(Y589_E611dup) mutation with a Variant Allele Frequency of 53%. For MPN1 sample, sctDNA-seq revealed that 91.91% of cells carried JAK2(V617F) mutation. For P293 MPN sample, bulk sequencing detected the presence of JAK2(V617F) mutation. AML1 and MPN12 patient cells were acquired from the Department of Internal Medicine I, Division of Hematology & Hemostaseology, Medical University of Vienna, Austria. P293 cells were provided by the Department of Hematology, Transplantation and Internal Medicine, University Clinical Care, Medical University of Warsaw, and the Department of Hematology, Institute of Hematology and Blood Transfusion, Warsaw, Poland. All further procedures were performed on Lin-CD34+ cells obtained as described before [35]. Patient cells were treated with 2 doses (Day 0, Day 3) of the indicated inhibitors and/or combinations for 6 days followed by Trypan blue exclusion test.

### Mouse bone marrow cells (mBMCs)

mBMCs were obtained and maintained as described before [36]. Lin-cKit+ wild-type and *Polq*^*−/−*^ cells were treated with either disulfiram (ALDH inhibitor) or N6022 (ADH5 inhibitor) for 48 h. Lin-cKit+ wild-type, *Adh5*^*−/−*^ and *Aldh2*^*−/−*^ cells were treated with ART558 (Polθ inhibitor) for 72 h. Clonogenic assay was performed as described before [36]. For replating assay, colonies were extracted from methylcellulose and replated in fresh methylcellulose.

### Detection of DPCs

Detection and quantification of DPCs was performed using the ARK assay as described before [10].

### Detection of DSBs

DSBs were detected by neutral comet assay, γH2AX immunofluorescence and western blot as described before [36] (Supplemental Materials and Methods).

### Formaldehyde detection

Formaldehyde detection of murine peripheral blood and bone marrow samples was performed using a formaldehyde detection kit, according to the manufacturer’s protocol (Abcam, ab196997).

### In vitro formaldehyde treatment

For DPC and γH2AX analysis, Lin-cKit+ mBMCs were treated with either 20 or 50 μM of formaldehyde for 12 h. For clonogenic assay, Lin-cKit+ mBMCs were treated with different concentrations of formaldehyde for 24 h and then plated in methylcellulose.

### In vivo methanol treatment

Methanol treatment protocol was previously described with some modifications [18]. In brief, *wt, Polq*^*−/−*^*, Adh5*^*−/−*^*, Aldh2*^*−/−*^*, Adh5*^*−/−*^*; Polq*^*−/−*^*, and Aldh2*^*−/−*^*; Polq*^*−/−*^ mice aged between 7- and 12-week old received intraperitoneal injections of methanol (Thermo Fisher Scientific #A412SK-4) on days 1 and 8. Methanol was diluted to 10% v/v in PBS, and injected at 12.6 mL kg^−1^. Weight was monitored daily and peripheral blood and bones were harvested on day 10 for analysis.

### Xenograft model

2 × 10^4^ Nalm6-*RAD54*^*−/−*^ human leukemia cells were injected into SCID Beige mice (Charles River, strain 250) via tail vein. Mice were then randomly assigned to 6 groups: control, disulfiram (ALDHi), N6022 (ADH5i), RP-6685 (Polθi), disulfiram (ALDHi) + RP-6685 (Polθi) and N6022 (ADH5i) + RP-6685 (Polθi). After 7 days, mice were treated via oral gavage daily for 14 days. In the groups where two treatments were combined, the drugs were administered at least 6 h apart. Both N6022 and disulfiram were dissolved in DMSO and further diluted using 10% Kolliphor HS 15 (Millipore #42966) and were administered to mice at 50 mg/kg/day. RP-6685 was first dissolved in DMSO and then in sterile water + 5% 1-methyl-2-pyrrolidinone (Millipore #443778) and 10% D-α-tocopherol polyethylene glycol 1000 succinate (Millipore #57668). RP-6685 was administered at 80 mg/kg/day. On day 23, 48 h after the last day of treatment, blood samples were collected via tail nick and an anti-human CD19 antibody (BD Biosciences #555413) was used to detect human CD19^+^ cells to determine leukemia burden using flow cytometry. Median survival time (MST) was determined as well.

### Hematological analyses

A Hemavet HV950 Multispecies Hematology Instrument (Drew Scientific) was used to perform automated blood cell counts from ~25 μL of blood. For immunophenotyping/flow cytometry analysis, please see Supplementary Materials and Methods.

### Western blot

Primary antibodies Polθ (MyBioSource #MBS9612322), ADH5 (Abcam #ab177932), ALDH2 (Invitrogen #MA5-17029), and actin (Invitrogen #MA5-11769) were used to detect these proteins in total cell lysates from mBMCs. Actin was used as a loading control.

### Viral infection

FLT3(ITD)-GFP or JAK2(V617F)-GFP were expressed in Lin-cKit+ mBMCs by viral infections as described before [[36] and Supplemental Materials and Methods].

### Inhibitors

Disulfiram (#S1680), N6022 (#S7589), ART558 (#S9936), RP-6685 (#E1528), ruxolitinib (#S1378) and quizartinib (#S1526) were all purchased from SelleckChem.

### ALDH and ADH5 activity

Wild-type and *Polq*^*−/−*^ Lin-ckit+ cells (1 × 10^6^ cells/well) were treated with various doses of disulfiram or N6022. After treatment, cells were pelleted and ALDH or ADH activity were measured according to protocols from ALDEFLUOR™ ALDH Detection Kit (StemCell Technologies #01700) and Alcohol Dehydrogenase Activity Colorimetric Assay Kit (BioVision company #K787), respectively.

### TMEJ activity assay

TMEJ activity assay was performed as describe before [10, 37]. Briefly, mBMCs from different mice were pretreated with ART558 (12.5 μM or 25 μM) for 24 h and co-transfected with pBABE-MMEJ plasmid [38] after digestion with I-SceI and dsRED-Mito (control for transfection efficiency) using Nucleofector^TM^ kit (Lonza VPA-1003). GFP^+^ and Red^+^ cells were detected after 72 h by BD Symphony A5 Analyzer. TMEJ activity is presented as the ratio of GFP^+^/Red^+^ cells.

### Ethics approval and consent to participate

All methods were performed in accordance with the relevant guidelines and regulations. All mouse experiments were approved by the Institutional Animal Care and Use Committees review board at Temple University. Studies involving patient samples at Temple University were not research involving human subjects as defined by the Department of Health and Human Services or Federal Drug Administration regulations. The patient samples were collected after obtaining informed consent in concordance with the Declaration of Helsinki and were approved by the institutional review boards.

## Results

### Polθ does not cooperate with ADH5 and ALDH2 to protect hematopoietic cells from the physiological levels of formaldehyde

To determine whether ADH5 and/or ALDH2 work together with Polθ to protect hematopoietic cells from physiological concentrations of formaldehyde, we crossed single-knockout *Polq*^*−/−*^, *Adh5*^*−/−*^, and *Aldh2*^*−/−*^ mice to generate double-knockout *Adh5*^*−/−*^*; Polq*^*−/−*^ and *Aldh2*^*−/−*^*; Polq*^*−/−*^ mice (Supplementary Fig. S1). These double knockout mice were viable and displayed no detectable growth abnormalities or any organ dysfunctions (Supplementary Fig. S2). *Adh5*^*−/−*^*; Aldh2*^*−/−*^*; Polq*^*−/−*^ mice could not be generated because most *Adh5*^*−/−*^*; Aldh2*^*−/−*^ mice die perinatally [18, 20].

**Fig. 1:**
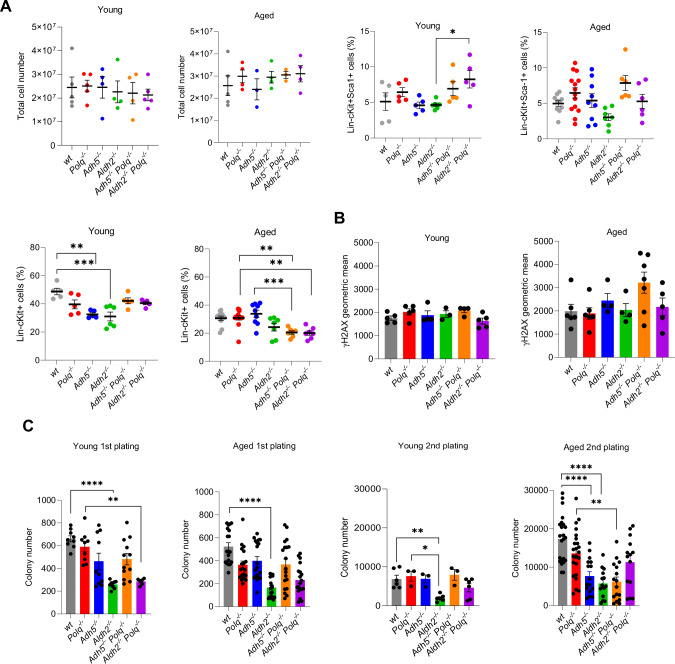
Polθ and ADH5 or ALDH2 do not cooperate to protect hematopoietic cells from physiological levels of formaldehyde. **A** Bone marrow cellular parameters: total mBMC counts from 2 femurs/mouse, percentage (%) of Lin-cKit+Sca-1+ and Lin-cKit+ cells in the bone marrow of the indicated genotypes of young (3-4 months old) and aged (>18 months old) mice. **B** γH2AX immunofluorescence in Lin-cKit+ BMCs from the indicated genotypes of mice. **C** Clonogenic activity of Lin-cKit+ BMCs from the indicated genotypes of mice tested in 1st and 2nd plating in methylcellulose. 2nd plating results represent a cumulative number of colonies to be formed if all cells from 1st plating were included. Each dot represents a result from an individual mouse. Results represent mean ± SEM. Statistical analysis was calculated using one-way ANOVA and Tukey’s test for multiple comparisons where *p ≦ 0.05, **p ≦ 0.01, ***p ≦ 0.001, ****p ≦ 0.0001.

To test the impact of the physiological levels of formaldehyde on hematopoiesis, hematological parameters were examined in young (3-4 months old) and aged (18-24 months old) wild-type, *Polq*^*−/−*^, *Adh5*^*−/−*^, *Aldh2*^*−/−*^, *Adh5*^*−/−*^*; Polq*^*−/−*^ and *Aldh2*^*−/−*^*; Polq*^*−/−*^ mice. We did not detect any significant changes in hematological blood parameters in young and aged double knockout mice when compared to both single-knockouts except for modestly elevated levels of Gr-1+Mac-1+ cells in young *Adh5*^*−/−*^*; Polq*^*−/−*^ mice (Supplementary Fig. S3). Similarly, we did not observe any major changes in bone marrow cells lineages of the double-knockout mice when compared to both single-knockouts except a modest decrease of the percentage of Lin-cKit+ HSPCs in aged *Adh5*^*−/−*^*; Polq*^*−/−*^ mice (Fig. 1A). The absence of Polθ and ADH5 or ALDH2 was also not associated with significant accumulation of DSBs measured by detection of γH2AX in Lin-cKit+ HSPCs (Fig. 1B). Moreover, serial clonogenic assays did not detect significant differences when comparing double-knockouts to single-knockouts in young and aged mice (Fig. 1C).

Overall, simultaneous knockouts of *Polq* and *Adh5* or *Aldh2* did not cause major abnormalities in hematological parameters of young and aged mice. Therefore, it seems unlikely that Polθ is needed to support ADH5 or ALDH2 in protecting murine hematopoietic cells from physiological levels of formaldehyde.

### Polθ cooperates with ADH5 and ALDH2 to protect hematopoietic cells challenged by high levels of formaldehyde

**Fig. 2:**
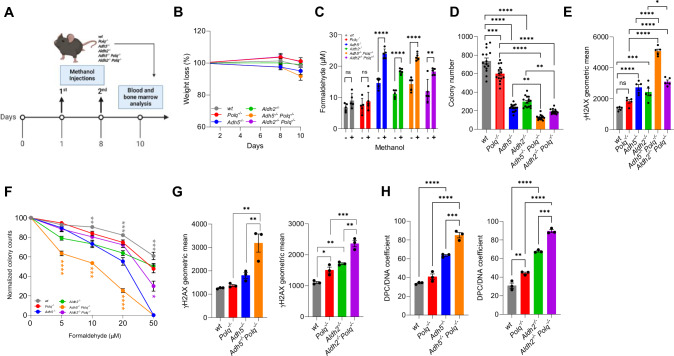
Polθ and ADH5 or ALDH2 cooperate to protect hematopoietic cells from formaldehyde challenge. **A**–**E** In vivo formaldehyde challenge. **A** Experimental timeline for in vivo methanol exposure, followed by analysis of the blood and bone marrow parameters. Created using Biorender.com. **B** Mean weights of mice ± SEM. **C** Mean formaldehyde levels ± SEM in peripheral blood of mice treated (+) or not (−) with methanol. **D** Mean number ± SEM of colonies formed by Lin-cKit+ BMCs. **E** Mean ± SEM of γH2AX immunofluorescence in Lin-cKit+ BMCs. **F**–**H** In vitro formaldehyde challenge. **F** Mean % of colonies ± SEM formed by Lin-cKit+ BMCs treated with various concentrations of formaldehyde. Results from the double knockouts were compared to those with the corresponding single knockouts, and results from wild-type were compared to the single knockouts. **G**, **H** Lin-cKit+ BMCs were treated with 20 μM (left) or 50 μM (right) of formaldehyde for 12 h. **G** Mean ± SEM of γH2AX immunofluorescence. **H** Mean level ± SEM of DPCs. Statistical significance was calculated using one-way ANOVA and Tukey’s test for multiple comparisons: *p ≦ 0.05, **p ≦ 0.01, ***p ≦ 0.001, ****p ≦ 0.0001. Each dot represents a result from an individual mouse.

Next, we examined if Polθ and ADH5 or ALDH2 protect hematopoietic cells from mice challenged by elevated levels of formaldehyde. For this purpose, young wild-type, *Polq*^*−/−*^, *Adh5*^*−/−*^, *Aldh2*^*−/−*^, *Adh5*^*−/−*^*; Polq*^*−/−*^ and *Aldh2*^*−/−*^*; Polq*^*−/−*^ mice were repeatedly injected with methanol (Fig. 2A) as previously described [18]. Methanol is an exogenous source of formaldehyde due to its oxidation by catalase and alcohol dehydrogenases 1 and 2, generating formaldehyde [2]. Methanol treatment did not cause any major weight loss (Fig. 2B). As expected, higher formaldehyde concentrations were detected in peripheral blood samples from *Adh5*^*−/−*^, *Aldh2*^*−/−*^, *Adh5*^*−/−*^*; Polq*^*−/−*^ and *Aldh2*^*−/−*^*; Polq*^*−/−*^ mice prior to methanol injections when compared to wild-type and *Polq*^*−/−*^ counterparts (Fig. 2C). Marked increases in formaldehyde levels were observed in *Adh5*^*−/−*^ and *Aldh2*^*−/−*^ ± *Polq*^*−/−*^ mice after administration of methanol (Fig. 2C) which were in the range detected in human blood [39]. However, the intracellular concentrations of formaldehyde within the bone marrow prior to and after methanol injections were unknown and are a limitation of this study.

The methanol derived formaldehyde did not alter the basic peripheral blood parameters in *Adh5*^*−/−*^*; Polq*^*−/−*^ and *Aldh2*^*−/−*^*; Polq*^*−/−*^ mice when compared to the corresponding single-knockouts, but seemed to enhance the percentage of Lin-cKit+Sca-1+ HSPCs in bone marrow of *Aldh2*^*−/−*^*; Polq*^*−/−*^ mice when compared to the single-knockouts (Supplementary Fig. S4). Remarkably, clonogenic capacity of Lin-cKit+ mBMCs obtained from methanol-treated *Adh5*^*−/−*^*; Polq*^*−/−*^ and *Aldh2*^*−/−*^*; Polq*^*−/−*^ mice was reduced when compared to that of the single knockouts (Fig. 2D) which was associated with accumulation of DSBs detected by γH2AX immunofluorescence (Fig. 2E). These results suggest the cooperation between Polθ and ADH5 or ALDH2 in protecting murine hematopoietic cells from formaldehyde challenge.

To further validate the role of Polθ and ADH5 or ALDH2 in protecting of HSPCs from the elevated concentrations of formaldehyde, wild-type, *Polq*^*−/−*^, *Adh5*^*−/−*^, *Aldh2*^*−/−*^, *Adh5*^*−/−*^*; Polq*^*−/−*^ and *Aldh2*^*−/−*^*; Polq*^*−/−*^ Lin-cKit+ mBMCs were incubated for 24 h with various concentrations of formaldehyde, followed by clonogenic assay. *Adh5*^*−/−*^*; Polq*^*−/−*^ HSPCs displayed higher sensitivity to the 5–20 μM formaldehyde range when compared to *Polq*^*−/−*^ and Adh*5*^*−/−*^ cells while *Aldh2*^*−/−*^*; Polq*^*−/−*^ cells demonstrated enhanced sensitivity only to 50 μM of formaldehyde when compared to *Polq*^*−/−*^ and Aldh*2*^*−/−*^ cells (Fig. 2F). Furthermore, the inhibition of the clonogenic activity of *Adh5*^*−/−*^*; Polq*^*−/−*^ and *Aldh2*^*−/−*^*; Polq*^*−/−*^ cells exposed to high-dose formaldehyde was also associated with enhanced accumulation of DSBs and DPCs (Fig. 2G and H, respectively). The higher formaldehyde sensitivity of *Adh5*^*−/−*^*; Polq*^*−/−*^ cells when compared to *Aldh2*^*−/−*^*; Polq*^*−/−*^ counterparts likely stems from ADH5 being the primary enzyme in formaldehyde detoxification, while ALDH2 has been described as a backup [18, 20].

Formaldehyde reacts with glutathione (GSH) followed by ADH5-mediated metabolism of formaldehyde-GSH products [20, 40]. GSH inhibitor L-buthionine sulfonimine (L-BSO) did not increase the sensitivity of *Adh5*^*−/−*^*; Polq*^*−/−*^ cells to formaldehyde-induced DPC-mediated DSBs but greatly enhanced the sensitivity of *Aldh2*^*−/−*^*; Polq*^*−/−*^ cells (Supplementary Fig. S5).

Altogether, these results clearly indicate that Polθ provides an additional tier of protection against elevated levels of formaldehyde in hematopoietic cells.

### Pharmacological inhibition of ADH5 or ALDH2 dehydrogenases elevates formaldehyde concentration and reduces the growth of *Polq*^*−/−*^ HSPCs

**Fig. 3:**
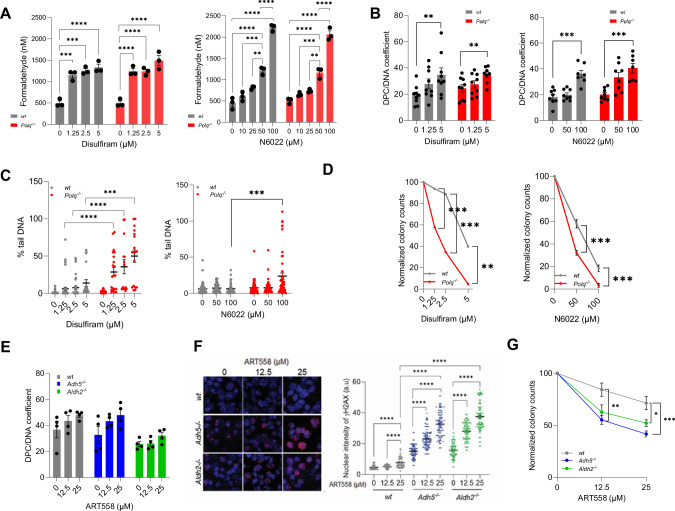
Simultaneous inhibition of Polθ and ADH5 or ALDH2 is toxic for HSPCs. **A**–**D** Wild-type and *Polq*^*−/−*^ Lin-cKit+ BMCs were treated with the indicated concentrations of disulfiram (ALDHi) or N6022 (ADH5i) for 48 h. **A** Mean ± SEM of formaldehyde levels. **B** Mean level ± SEM of DPCs. **C** Mean % ± SEM of tail DNA from neutral comet assay. **D** Mean % ± SEM of colonies when compared to untreated counterparts. **E**–**G** Wild-type, *Adh5*^*−/−*^ and *Aldh2*^*−/−*^ Lin-cKit+ BMCs were treated with the indicated concentrations of ART558 (Polθi) for 72 h. **E** Mean level ± SEM of DPCs. **F** Representative γH2AX nuclear foci (red), DNA was counterstained with DAPI (blue) (left panel). Mean level ± SEM of the γH2AX nuclear foci intensity (right panel). **G** Mean % ± SEM of colonies when compared to untreated counterparts. Statistical significance was calculated using one-way ANOVA and Tukey’s test for multiple comparisons; *p ≦ 0.05, **p ≦ 0.01, ***p ≦ 0.001, ****p ≦ 0.0001.

Murine HSPCs carrying constitutive *Polq* knockout combined with *Adh5* or *Aldh2* knockout might develop compensatory mechanisms masking genuine biological roles of these dehydrogenases. Therefore, to validate the collaboration between Polθ and ADH5 or ALDH2 in protecting HSPCs from formaldehyde challenge, ADH5 and ALDH2 dehydrogenase activities were inhibited by ADH5 inhibitor (ADH5i) N6022 or ALDH inhibitor (ALDHi) disulfiram, respectively, in wild-type and *Polq*^*−/−*^ Lin^-^cKit^+^ HSPCs (Supplementary Fig. S6A) [41, 42]. Inhibition of ADH5 or ALDH2 resulted in accumulation of formaldehyde (Fig. 3A) and DPCs (Fig. 3B) in both wildtype and *Polq*^*−/−*^ cells. However, neutral comet assay revealed enhanced DSBs (Fig. 3C, Supplementary Fig. S6B) and reduced clonogenic potential (Fig. 3D) only in *Polq*^*−/−*^ HSPCs treated with N6022 and disulfiram when compared to wild-type counterparts.

Furthermore, inhibition of Polθ -mediated TMEJ by the polymerase inhibitor ART558 [43] (Supplementary Fig. S6C) did not significantly increase DPCs in *Adh5*^*−/−*^ and *Aldh2*^*−/−*^ Lin^-^cKit^+^ HSPCs (Fig. 3E), but caused accumulation of DSBs (Fig. 3F, Supplementary Fig. S6D, E), consistent with the role of Polθ in repairing DSBs, but not DPCs. The increase in DSBs was associated with reduced colony formation of *Adh5*^*−/−*^ and *Aldh2*^*−/−*^ HSPCs treated with ART558 when compared to wild-type counterparts (Fig. 3G).

Since formaldehyde also triggers oxidative DNA lesions, we tested the role of Polθ in responding to oxidative DNA damage. Remarkably, *Polq*^*−/−*^ HSPCs displayed similar sensitivity to hydrogen peroxide and accumulated similar amounts of reactive oxygen species (ROS), oxidative DNA damage (8-oxoG) and DSBs as the wild-type cells (Supplementary Fig. S7). This observation highlights the collaborative role of Polθ and ADH5/ADLH2 in selective protection of HSPCs against the toxic effect of formaldehyde-mediated DSBs, but not hydrogen peroxide-mediated DSBs in HSPCs.

### Polθ and ADH5 or ALDH2 protect AML and MPN cells from genotoxic effect of formaldehyde induced by OTKs

**Fig. 4:**
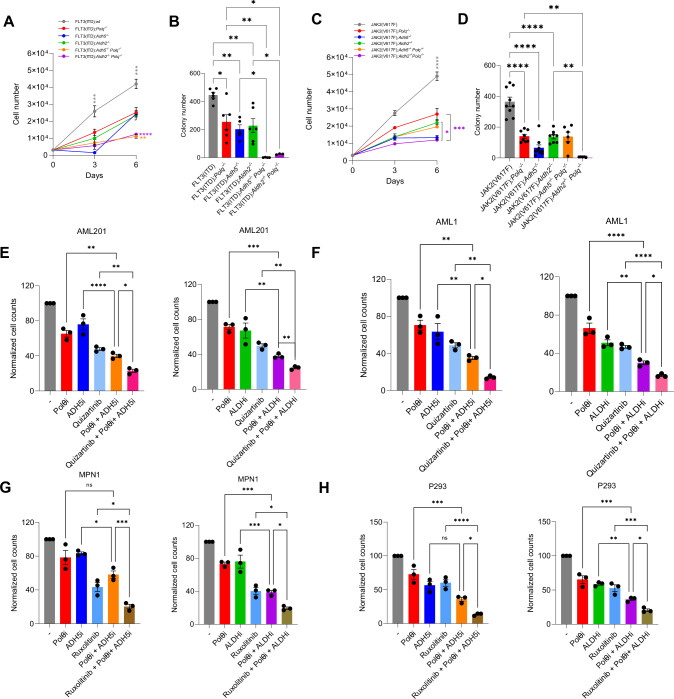
Polθ and ADH5 or ALDH2 protect malignant HSPCs from formaldehyde challenge triggered by OTKs. **A**, **B** Proliferation and clonogenic activity of FLT3(ITD)-positive *wt*, *Polq*^*−/−*^, *Adh5*^*−/−*^, *Aldh2*^*−/−*^, *Adh5*^*−/−*^*; Polq*^*−/−*^ and *Aldh2*^*−/−*^*; Polq*^*−/−*^ Lin-cKit+GFP+ BMCs. **A** Mean number ± SEM of cells in liquid culture. **B** Mean number ± SEM of colonies. **C**, **D** Proliferation and clonogenic activity of JAK2(V617F)-positive *wt*, *Polq*^*−/−*^, *Adh5*^*−/−*^, *Aldh2*^*−/−*^, *Adh5*^*−/−*^*; Polq*^*−/−*^ and *Aldh2*^*−/−*^*; Polq*^*−/−*^ Lin-cKit+GFP+ BMCs. **C** Mean number ± SEM of cells in liquid culture. **D** Mean number ± SEM of colonies. **E** Mean number ± SEM of FLT3(ITD)-positive AML 201 patient cells treated with 25 μM Polθi, 0.075 μM ALDHi, 75 μM ADH5i, 0.125 μM FLT3i alone or in combinations for 6 days. **F** Mean number ± SEM of FLT3(ITD)-positive AML1 patient cells treated with 25 μM Polθi, 0.075 μM ALDHi, 75 μM ADH5i, 0.125 μM FLT3i alone or in combinations for 6 days. **G** Mean number ± SEM of JAK2(V617F)-positive MPN1 patient cells treated with 25 μM Polθi, 0.05 μM ALDHi, 50 μM ADH5i, 0.1 μM JAK1/2i alone or in combinations for 6 days. **H** Mean number ± SEM of JAK2(V617F)-positive MPN P293 patient cells treated with 25 μM Polθi, 0.075 μM ALDHi, 75 μM ADH5i, 0.1 μM JAK1/2i alone or in combinations for 6 days. Statistical significance was calculated using one-way ANOVA and Tukey’s test for multiple comparisons; *p ≦ 0.05, **p ≦ 0.01, ***p ≦ 0.001, ****p ≦ 0.0001.

We have recently reported that OTKs such as FLT3(ITD) and JAK2(V617F) stimulate one-carbon metabolism, resulting in accumulation of the high levels of formaldehyde and that Polθ plays a critical role in protecting AML and MPN cells from the genotoxic effect of formaldehyde [10]. To test if Polθ collaborates with ADH5 and/or ALDH2 in protecting the leukemic cells from formaldehyde, FLT3(ITD) was expressed in Lin-cKit+ mBMCs from wild-type, *Polq*^*−/−*^*, Adh5*^*−/−*^, *Aldh2*^*−/−*^, *Adh5*^*−/−*^*; Polq*^*−/−*^ and *Aldh2*^*−/−*^*; Polq*^*−/−*^ mice. We found that proliferation and colony formation by FLT3(ITD)-positive double-knockout *Adh5*^*−/−*^*; Polq*^*−/−*^ and *Aldh2*^*−/−*^*; Polq*^*−/−*^ cells were severely reduced when compared to FLT3(ITD)-positive single-knockout *Polq*^*−/−*^, *Adh5*^*−/−*^, and *Aldh2*^*−/−*^ counterparts (Fig. 4A, B), indicating combined protective effect of Polθ with either of these dehydrogenases. In addition, clonogenic capacity of JAK2(V617F)-positive *Aldh2*^*−/−*^*; Polq*^*−/−*^ double-knockout Lin-cKit+ mBMCs was also almost completely suppressed when compared to *Aldh2*^*−/−*^ and *Polq*^*−/−*^ counterparts (Fig. 4C, D). However, in contrast to FLT3(ITD), proliferation and clonogenicity of JAK2(V617F)-positive *Adh5*^*−/−*^*; Polq*^*−/−*^ cells was like that of *Adh5*^*−/−*^ and *Polq*^*−/−*^ counterparts suggesting that Polθ and ADH5 do not functionally cooperate in JAK2(V617F)-positive HSPCs.

In concordance with the genetic knockout data (Fig. 4A–D), FLT3(ITD)-positive AML 201 and AML1 patient cells (Fig. 4E, F) and JAK2(V617F)-positive MPN1 and P293 MPN patient cells (Fig. 4G, H) were more sensitive to the combination of ALDHi and Polθi when compared to treatment with these drugs used as single agents. As with murine HSPCs, only FLT3(ITD)-positive AML primary cells, but not JAK2(V617F)-positive MPN primary cells were more sensitive to ADH5i and Polθi. Noteworthy, FLT3i quizartinib and JAK1/2i ruxolitinib enhanced the effect of the combinations of Polθi + ALDHi and Polθi + ADH5i in AML and MPN patient cells.

These data indicate that Polθ cooperates with ALDH2 and/or ADH5 in protecting AML and MPN cells from the genotoxic effect of formaldehyde. This statement is also indirectly supported by the results of TCGA dataset mining showing that while the mRNA expression of *ALDH2* and *ADH5* is relatively low in AML when compared to other tumors, it seemed to be compensated by the highest level of *POLQ* mRNA expression (Supplementary Fig. S8A). In addition, *POLQ* and *ADH5/ALDH2* genetic aberrations appeared mutually exclusive in tumors in the TCGA datasets (Supplementary Fig. S8B) suggesting that they belong to the cooperating oncogenic pathways.

### Simultaneous inhibition of Polθ and ADH5 or ALDH2 exerts anti-leukemia effect

Considering that inhibition of Polθ is synthetically lethal in HR-deficient malignant cells [44, 45], we speculated that inhibition of ADH5 and/or ALDH2 may enhance this anti-tumor effect. To test this hypothesis, we employed human Nalm6 parental leukemic cells and their isogenic HR-deficient *RAD54* knockout counterparts; the latter displaying hypersensitivity to Polθi [10, 46]. The cells were treated with ART558 (Polθi), N6022 (ADH5i) and disulfiram (ALDHi) either alone or in combination.

**Fig. 5:**
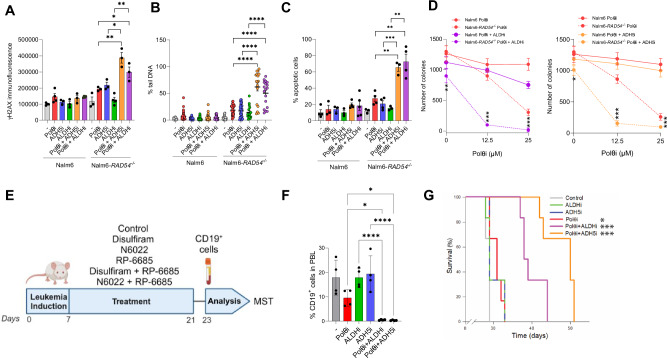
Combination of Polθi + ALDHi and Polθi + ADH5i increased DNA damage and abrogated the proliferation of HR-deficient leukemia cells. **A**–**C** Nalm6 and Nalm6-*RAD54*^−/−^ cells were untreated (−) or treated with 12.5 μM Polθi, 0.125 μM ALDHi, 20 μM ADH5i, and the indicated combinations for 48 h. **A** Mean ± SEM of γH2AX immunofluorescence. **B** Mean % tail DNA ± SEM from neutral comet assay. **C** Mean % ± SEM of the annexin V/propidium iodide double-stained cells. **D** Nalm6 and Nalm6-*RAD54*^*−/−*^ cells were treated with 12.5 μM or 25 μM of Polθi ± 0.125 μM ALDHi (*left*) and 12.5 μM or 25 μM of Polθi ± 20 μM ADH5i (*right*) for 48 h followed by clonogenic assay. Results represent mean % of colonies ± SEM when compared to untreated counterparts. **E** Experimental timeline for mice that were injected with Nalm6-*RAD54*^*−/−*^ cells. Created using Biorender.com. **F** Percentage of CD19^+^ cells detected in the peripheral blood of mice on day 23. **G** Survival plots of the mice treated with the indicated inhibitors. Statistical significance was calculated using: (**A**–**D**, **F**) one-way ANOVA and Tukey’s test for multiple comparisons; *p ≦ 0.05, **p ≦ 0.01, ***p ≦ 0.001, ****p ≦ 0.0001, and **H** Kaplan–Meier Log-Rank survival analysis; ***p ≦ 0.001 (versus individual treatment), and *p ≦ 0.05 (versus Control).

Combined with Polθi, either ADH5i or ALDHi enhanced accumulation of DSBs selectively in HR-deficient Nalm6-*RAD54*^*−/−*^ cells (Fig. 5A, B). Moreover, these double-inhibitor combinations increased the percentage of apoptotic cells by 3-4 -fold in Nalm6-*RAD54*^*−/−*^ cells, while exerting very modest effect in Nalm6 parental cells (Fig. 5C, Supplementary Fig. S9).

Accumulation of DSBs and induction of apoptosis in Nalm6-*RAD54*^*−/−*^ cells treated with Polθi and ADH5i or ALDHi was accompanied by reduction of their clonogenic capacity when compared to parental counterparts (Fig. 5D). For example, 0.125 μM disulfiram enhanced the effect of 12.5 μM ART558 by >6-fold and of 25 μM ART558 by >10-fold in Nalm6-*RAD54*^*−/−*^ cells, while exerting very modest effect in Nalm6 counterparts (Fig. 5D). Similarly, 20 μM N6022 enhanced the effect of 12.5 μM ART558 by >4-fold and of 25 μM ART558 by >2-fold in Nalm6-*RAD54*^*−/−*^ cells, while no effect was observed in Nalm6 parental cells (Fig. 5D). Based on these findings, we postulate that ADH5 and ALDH2 inhibitors enhance the synthetically lethal effect of Polθi against HR-deficient leukemia cells.

To test if the combinations of Polθi and ADH5i or ALDHi may have potential clinical applications, SCID-beige mice bearing Nalm6-*RAD54*^*−/−*^ leukemia were treated with RP-6685, a potent, selective, and orally bioavailable Polθi [47] alone and in combination with disulfiram or N6022 for 14 consecutive days (Fig. 5E). Two days after the end of treatment, peripheral blood was examined for the presence of human CD19^+^ cells. The combination of Polθi and ADH5i or ALDHi reduced the percentage of CD19^+^ Nalm6-*RAD54*^*−/−*^ leukemia >10x when compared to single treatment (Fig. 5F).

Leukemia-bearing control, ALDHi and ADHi -treated mice succumbed to leukemia after 28.7 ± 0.2, 30.2 ± 0.9 and 30.3 ± 0.8 days, respectively (Fig. 5G). Administration of Polθi caused only a very modest increase of median survival time (MST) (30.8 ± 0.8 days). Remarkably, combinations of Polθi + ADH5i and Polθi + ALDHi resulted in substantial elongations of MST (47.8 ± 1.7 and 40.0 ± 1.3 days, respectively) when compared to individual treatments. Polθi + ADH5i exerted significantly stronger anti-leukemia effect when compared to Polθi + ALDHi (*p* < 0.02). All mice succumbed to leukemia and harbored 16.4 ± 6.7% and 27.0 ± 15.0% of CD19^+^ cells in peripheral blood and bone marrow, respectively.

## Discussion

Formaldehyde is ubiquitous within cells and is endogenously generated throughout our lives. ADH5 and ALDH2 protect cells from these physiological, yet otherwise lethal concentrations of formaldehyde that can create genome instability and overwhelm DNA repair pathways [3, 18–20]. Therefore, formaldehyde could be a major mutagen in the mammalian genome where highly efficient protection systems may be important not only in the context of bone marrow failure syndromes and leukemia, but also in other cancers [3]. In concordance, ALDH2 deficiency has been observed to affect the risk of carcinogenesis [21–24]. Studies have also shown that formaldehyde plays a causal role in the development of nasopharyngeal cancers [4].

Protection against formaldehyde is essential due to its ability to chemically react with DNA to create covalent crosslinks between DNA and/or proteins. HSPCs are consistently exposed to physiological levels of formaldehyde, generating potentially lethal DNA lesions such as ICLs and DPCs [1–3]. To promote cell survival and maintain genome stability, HSPCs rely on a two-tiered protection system. ADH5 and ALDH2 metabolize formaldehyde (Tier-1) while the FA pathway repairs potentially lethal formaldehyde-induced ICLs (Tier-2) [2, 3, 19]. DPCs acquired under physiological levels of formaldehyde are likely resolved by SPRTN protease and TDP1/2 hydrolases before generating DSBs [48].

**Fig. 6:**
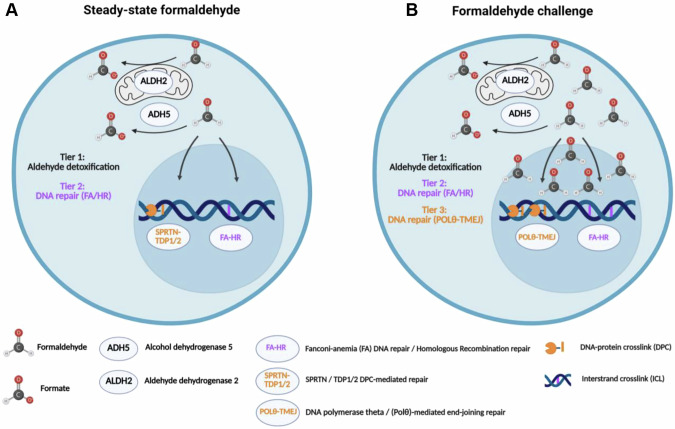
Polθ collaborates with ADH5 and ALDH2 to protect normal and malignant HSPCs against formaldehyde challenge. **A** Under physiological conditions, HSPCs are protected against formaldehyde by a previously described “2-Tier” system: ADH5 and ALDH2 detoxify formaldehyde into the less reactive metabolite formate (1^st^ tier of protection) while the FA DNA repair pathway repairs ICLs (2^nd^ tier of protection). Infrequent DPCs are digested by SPRTN and TDP1/2. **B** Newly described “3-Tier” system where HSPCs challenged by elevated levels of formaldehyde accumulate not only ICLs but also DPC-induced toxic DSBs which are repaired by Polθ-mediated TMEJ (3^rd^ tier). Created using Biorender.com.

Here, we report that Polθ provides a Tier-3 protection layer in normal and malignant HSPCs challenged by elevated levels of formaldehyde generated by extracellular or intracellular sources (Fig. 6). Thus, we postulate that while Tier-1/Tier-2 are largely sufficient to protect HSPCs exposed to physiological levels of formaldehyde, Tier-3 becomes critical when the FA pathway is overwhelmed by excessive DNA damage triggered by higher levels of formaldehyde [1, 3, 4, 9, 10]. The protective effect of Polθ most likely depends on its ability to repair 5’-DPC-mediated DSBs which are produced by MRN-CtIP pathway initiating endonucleolytic cleavage of DPCs at DSBs, followed by TMEJ [27, 49]. Formaldehyde-induced DPCs can stall replication forks, leading to potential fork collapse and DSBs [50]. In addition to DPCs, formaldehyde can induce other DNA lesions, such as single-strand breaks and base modifications, which can also contribute to DSBs formation [51]. We reported that Polθ-TMEJ can promote DSB repair which depends or not on replication forks [27].

The existence of Tier-1 (ADH5/ALDH2), Tier-2 (FA pathway) and Tier-3 (Polθ) layers protecting hematopoietic cells against basal and/or high levels of formaldehyde is supported by genetic studies. For example, simultaneous knockout of Tier-1 (*Adh5*^*−/−*^) and Tier-2 (*Fancd2*^*−/−*^) members was lethal in mice supporting the statement that Tier-1 + Tier-2 protect cells from the basal levels of formaldehyde. However, mice bearing the genetic knockout of Tier-1 (*Adh5*^*−/−*^) and Tier-3 (*Polq*^*−/−*^) members were viable, and the toxicity was detected only when hematopoietic cells were challenged with elevated levels of formaldehyde (this work).

In addition to FA and Polθ, exonuclease 1 (EXO1) has been identified to remove exogenous formaldehyde-triggered DPCs and ICLs and to promote cell survival of epithelial cells [52]. Moreover, ADH5 and Cocayne syndrome B (CSB, a member of the transcription-coupled nucleotide excision repair = TC-NER) provide Tier-1 and Tier-2 protection, respectively, against DPCs and ICLs caused by endogenous formaldehyde in the brain and kidney cells [8, 51, 53, 54] whereas ADH5 and FA protect the liver, kidney and hematopoietic cells [2]. Altogether, it appears that cells are equipped with organ-specific multi-tiered mechanisms protecting them from formaldehyde toxicity [2, 8, 18, 19, 48, 49, 52, 55].

We postulate that the interdependence between Polθ and ALDH2/ADH5 in protecting leukemia cells from high levels of endogenous formaldehyde creates a translational opportunity [56, 57]. Our studies suggest that ALDHi [disulfiram, FDA-approved drug for the treatment of alcoholism [41] and/or ADH5i [N6022, evaluated against pulmonary emphysema and asthma [58, 59] should enhance the anti-leukemic effect of Polθi in cohorts of HR-deficient acute leukemias and in OTK-positive myeloid malignancies (AML and MPN) which accumulate high levels of endogenously produced formaldehyde [10, 35]. Thus, clinical application of the Polθi and ADH5i/ALDHi combination deserves consideration.

## Supplementary information

Supplemental Materials and Results

## Data Availability

Data sharing not applicable to this article as no datasets were generated or analyzed during the current study.
