# Supplementary material for: ADH5/ALDH2 dehydrogenases and DNA polymerase theta protect normal and malignant hematopoietic cells from formaldehyde challenge: therapeutic implications
Source: Leukemia. 2025 Jul 10;39(9):2152–62. doi: 10.1038/s41375-025-02687-3 (PMC12380611; doi:10.1038/s41375-025-02687-3)
Supplement: Supplementary file 1 — Supplemental Materials and Results [file 41375_2025_2687_MOESM1_ESM.pdf]

Mouse genotyping

All mice were identified by polymerase chain reaction (PCR) by a tail snip of DNA from mice. DNA isolation and purification from mouse tails was performed using the following protocol. 240 µL of 50 mM NaOH was added to each tissue sample and then heated at 95°C for 10-20 minutes with vortexing when using larger tail snip samples. Then, 60 µL of Tris HCl pH 8 1M was added to each sample, mixed, and centrifuged for 5 minutes at maximum speed. The ratio of NaOH and Tris HCl can be adjusted based on the size of the tail snip. Samples were stored long term at -20°C. Genotyping was performed using knockout specific primers (Eurofins) and 2X DreamTaq polymerase Master Mix (Thermo Fisher Scientific #K1081). *Polq*-specific primers were: wild-type forward: 5'-TGCAGTGTACAGATGTTACTTTT-3'; wild-type reverse: 5'-TGGAGGTAGCATTTCTTCTC-3'; mutant forward: 5'-TCACTAGGTTGGGGTTCTC-3'; mutant reverse: 5'-CATCAGAAGCTGACTCTAGAG-3'. Two separate PCR reactions were set up, WT primers 1 and 2 together and mutant primers 1 and 2 together. The primers amplified a 190 base pair fragment if wild-type, a 300 base pair fragment if *Polq* null, and both 190 and 300 base pair fragments if heterozygous. The amplification conditions for *Polq* specific primers consisted of one cycle at 94°C for 3 mins, followed by 34 cycles at 94°C for 30 sec, 60°C for 30 sec, and 68°C for 45 sec. Then, 68°C for 5 mins. *Adh5*-specific primers were: wild-type 5'-GAAATGAGCCTGCCCTTCCA-3', wild-type 5'-CTGTCATCTCAACGAGGACTTC-3', mutant 5'-CGCCTTCTATCGCCTTCTTGACGAGT-3' and mutant 5'-TTCTTCCCCTGAAGCAGCTA-3'. One reaction was set up combining all four primers together. The primers amplified a 600 bp fragment if wild type, 900 bp fragment if *Adh5* null, and both 600 bp and 900 bp fragment if heterozygous. The amplification conditions for *Adh5* specific primers consisted of one cycle at 95°C for 5 min, followed by 34 cycles at 95°C for 30 sec, 59°C for 30 sec, and 72°C for 1 min. Then, 72°C for 7 min. *Aldh2*-specific primers were as follows: ST316 Forward: 5'-AAACTTTGCACACACTGTCCC-3', ST317 Reverse 1: 5'-CCCAGATCCAACTGTTAGGAATAC-3', and ST318 Reverse 2: 5'-GCTTCACTGAGTCTCTGGCATCTC-3'. One reaction was set up combining all three primers together in a F:R1:R2=2:1:1 ratio. The primers amplified a 212 bp fragment if wild type, 414 bp fragment if *Aldh2* null and both 212 bp and 414 bp fragments if heterozygous. The amplification conditions for *Aldh2* specific primers consisted of one cycle at 95°C for 2 min, followed by 30 cycles at 95°C for 30 sec, 62°C for 30 sec, and 72°C for 30 sec. Then, 72°C for 5 min. All PCR products were run on a 2.0% agarose gel containing ethidium bromide and visualized using the iBright 1500 Imaging System (Invitrogen). GeneRuler 100 bp ladder was used (Thermo Fisher Scientific #SM0241).

Detection of DSBs

Comet assays were performed using the OxiSelect™ Comet Assay Kit (Cell Biolabs #STA-351) according to the manufacturer's instructions. Images were acquired by an inverted Olympus IX70 fluorescence microscope using a FITC filter, and the percentage of tail DNA of individual cells was calculated using the OpenComet plugin of ImageJ. For γH2AX immunofluorescence, cells were fixed with 2% PFA for 15-30 minutes on ice. Cells were then washed with 1x PBS and resuspended in 100 µL PBS and 1 mL of pre-chilled 100% methanol. Cells incubated for 15-30 minutes on ice, then spun down and washed twice with 0.02% Triton-X in PBS. After the second wash, cells were resuspended in 100 µL PBS with 1% BSA and 0.02% Triton-X. Antibody for γH2AX (BD Biosciences, 560447) was added and cells incubated for about 30 minutes at room temperature in the dark. After incubation, cells were washed and resuspended with PBS containing 0.02% Triton-X. Cells were then analyzed using the BD Symphony A5 Analyzer. Geometric mean was calculated for γH2AX using FlowJo software. For γH2AX western blot, anti-γH2AX antibody (Abcam Cat#: ab243906) was used.

γH2AX immunofluorescence (IF)

Cells were processed and analyzed for IF as described before (37). After ART558 treatment, cells were fixed with 4% paraformaldehyde for 20 minutes at 4°C, washed with PBS, followed by permeabilized with 0.5% Triton X-100 for 10 minutes and blocked with PBS containing 3% BSA for 1 hour. Cells were then incubated with the same buffer containing primary antibodies anti-γH2AX (Abcam Cat#: ab243906) at 1:100 overnight at 4°C followed by 1 hour incubation in the dark with secondary antibodies anti-mouse Alexa Fluor 594 (Life Technologies Cat#: A11062). 5x washing in PBST for 3 minutes after each antibody incubation. Slides were mounted in 20 µL mounting media. Cells were visualized and imaged using a Leica SP8 Confocal microscope at a 63X objective magnification and images were analyzed using ImageJ software. For quantification, > 30 cells were counted for all conditions from three independent experiments. Intensity of individual protein were analyzed in individual nuclei (N=30) by measuring the foci formation [as intensity (a.u)] using ImageJ.

Sensitivity to H_2O_2

Wild-type and *Polq*$^{-/-}$ mBMCs were treated with various doses of hydrogen peroxide (Thermo Fisher Scientific #H325-500) for 4 hours. After treatment, cells were collected and the following assays were performed: clonogenic assay, neutral comet assay, detection of ROS using 2′,7′-dichlorodihydrofluorescein diacetate (H2DCFDA) (Sigma Aldrich Cat#: D6883) and detection of 8-oxo-G by flow cytometry (Abcam Cat#: ab183393) using the same protocol as described above for γH2AX. For the detection of ROS, after hydrogen peroxide treatment, H2DCFDA dye was added to cells at a final concentration of 5 µM and kept in the dark at 37 °C for 30 minutes. Cells were then washed in PBS and analyzed by flow cytometry. Geometric mean was measured for both 8-oxo-G and H2DCFDA.

Detection of apoptosis

Flow cytometry and staining with propidium iodide and FITC Annexin V was used to assess changes in viability and to track the mechanism of cell death. Cells were prepared and analyzed according to the FITC Annexin Apoptosis Detection Kit II (BD Biosciences #556570).

Immunophenotyping/flow cytometry analysis

Analysis of mouse peripheral blood: Mouse blood samples were taken either via tail vein nick or cardiac puncture. Red blood cells (RBCs) were lysed using 5 mLs of ACK buffer and placed on ice for 5 minutes. The lysate was then spun down at approximately 1200 rpm for 5 minutes. Lysate was then washed twice with 1 mL PBS and spun at 1200 rpm for 5 minutes. Cells were then resuspended in 90 µL 1% BSA (Sigma-Aldrich #A9576) in PBS + 10 µL Fc blocker (Invitrogen #14-0161-86) for 10 minutes. Cells were then resuspended in PBS and antibodies for cell surface markers Gr-1 (BD Biosciences #557405), Mac-1 (BD Biosciences #553311), B220 (BD Biosciences #553092), and CD3 (BD Biosciences #552774) and incubated for 30 mins at 4ºC while protected from the light. Cells were washed with 3 mL of PBS and spun at 1200 rpm for 5 minutes to remove supernatant. Samples were then fixed with 2% PFA. Analysis of mBMCs: cells were depleted of erythroid cells by using 5 mL of ACK buffer and placed on ice for 5 minutes. The cells were then spun down at approximately 1000 rpm for 5 minutes. Cells were washed in 1x PBS and spun down again at 1000 rpm for 5 minutes. Then, cells were resuspended in 90 µL 1% BSA in PBS + 10 µL Fc blocker for 10 minutes. Cells were then resuspended in PBS and antibodies for cell surface markers Lin- (BD Biosciences #558074), cKit+ (BD Biosciences #553355), and Sca-1+ (BD Biosciences #558162) and incubated for 30 minutes at 4ºC away from

the light. Cells were then resuspended in 2% PFA. All samples were analyzed using the BD Symphony A5 Analyzer.

Murine organs extraction

All mice were euthanized by exposure to CO_2, followed by cervical dislocation. Immediately following euthanasia, organs were extracted to 4% paraformaldehyde (Thermo Fisher Scientific #J19943-K2) and analyzed as described before (46).

Viral infection

10 µg of the FLT3(ITD)-GFP or JAK2(V617F)-GFP plasmid and 5 µg pCL-Eco (Addgene, plasmid #12371) were co-transfected into Phoenix-ECO cells (ATCC #CRL-3214) in DMEM culture medium with Lipofectamin 2000 Transfection Reagent (Thermo Fisher Scientific #11668019). After 24 and 48 hours of the transfection, the retroviral supernatant was collected and purified using 0.45 µm filter. For transduction, 0.5-1 x 10^6 cells were resuspended in 1 mL retroviral supernatant with 8 µg polybrene (Millipore #TR-1003). Spin-infection was performed at 1800 rpm at 30ºC for 90 minutes in a 24-well plate. Cells were incubated for 4 hours or left overnight and replaced with regular medium. GFP^+ cells were selected by sorting via flow cytometry.

L-BSO sensitivity assay

1x10^4 cells/mL were seeded in 96-well plates. Cells were treated with the indicated concentrations of formaldehyde and/or 50 µM of L-Buthionine sulfoximine (L-BSO) (MedChem Express Cat#: HY-106376A) for 24 hours followed by plating in methylcellulose. Colonies were counted after 7 days.

STAR* METHODS

	SOURCE	IDENTIFIER
**Antibodies**		
**Anti-Actin**	Invitrogen	Cat#: MA5-11769
**Anti-POLθ**	MyBioSource	Cat#: MBS9612322
**Anti-ADH5**	Abcam	Cat#: ab177932
**Anti-ALDH2**	Invitrogen	Cat#: MA5-17029
**Anti-Human CD19**	BD Biosciences	Cat#: 555413
**Anti- γH2AX**	BD Biosciences	Cat#: 560447
**Anti- γH2AX**	Abcam	Cat#: ab243906
**Anti-Mouse Alexa Fluor 594**	Life Technologies	Cat#: A11062
**Anti-Rabbit (Secondary antibody)**	LICORbio	Cat#: 926-68073
**Anti-Goat (Secondary antibody)**	LICORbio	Cat#: 926-32210
**Anti-DNA/RNA Damage (8-Oxo-G)**	Abcam	Cat#: ab183393
**Anti-Mouse Ly-6A/E (Gr-1)**	BD Biosciences	Cat#: 557405
**Anti-Mouse CD11b (Mac-1)**	BD Biosciences	Cat#: 553311
**Anti-Mouse CD45R/B220**	BD Biosciences	Cat#: 553092
**Anti-Mouse CD3e**	BD Biosciences	Cat#: 552774
**Anti-Mouse CD117 (cKit+)**	BD Biosciences	Cat#: 553355
**Anti-Mouse Ly-6A/E (Sca-1+)**	BD Biosciences	Cat#: 558162
**Anti-Mouse CD16/CD32 (Fc block)**	Invitrogen	Cat#: 14-0161-86
**APC Mouse Lineage Antibody Cocktail**	BD Biosciences	Cat#: 558074
**Commercial Kits**		
**OxiSelect™ Comet Assay Kit (3-Well Slides)**	Cell Biolabs	Cat#: STA-351
**Formaldehyde Assay Kit**	Abcam	Cat#: ab196997
**2x DreamTaq Polymerase Master Mix**	Thermo Fisher Scientific	Cat#: K1081
**GeneRuler 100 bp ladder**	Thermo Fisher Scientific	Cat#: SM0241
**Mouse Direct Lineage Cell Depletion Kit**	Miltenyi Biotec	Cat#: 130-110-470
**Easy Sep™ Mouse CD117 (cKit+) Positive Selection Kit**	StemCell Technologies	Cat#: 18757
**EasySep™ Human Progenitor Cell Enrichment Kit with Platelet Depletion**	StemCell Technologies	Cat#: 19356

**EasySep™ Human CD34 Positive Selection Kit II**	StemCell Technologies	Cat#: 17856
**Nucleofector™ kit**	Lonza	Cat#: VPA-1003
**ALDEFLUOR™ ALDH Detection Kit**	StemCell Technologies	Cat#: 01700
**Alcohol Dehydrogenase Activity Colorimetric Assay Kit**	Biovision	Cat#: K787
**FITC Annexin Apoptosis Detection Kit II**	BD Biosciences	Cat#: 556570
**Cell lines**		
**Nalm6 parental**		Ref. 34
**Nalm6-*RAD54*$^{-/-}$**		Ref. 34
**Phoenix-ECO cells**	ATCC	Cat#: CRL-3214
**Reagents**		
**RPMI 1640 medium**	Genesee Scientific	Cat#: 25-506
**IMEM medium**	Corning	Cat#: 10-016-CV
**DMEM medium**	Genesee Scientific	Cat#: 25-500
**StemSpan SFEM medium**	StemCell Technologies	Cat#: 09655
**MethoCult™ H4230**	StemCell Technologies	Cat#: 04230
**Fetal Bovine Serum (FBS)**	Gibco	Cat#: 10437-028
**ACK lysis buffer**	Thermo Fisher Scientific	Cat#: A1049201
**Antibiotic Antimycotic Solution 100x**	Corning	Cat#: 30-004-CI
**Methanol**	Thermo Fisher Scientific	Cat#: A412SK-4
**Formaldehyde**	Thermo Fisher Scientific	Cat#: F79-500
**Paraformaldehyde**	Thermo Fisher Scientific	Cat#: J19943-K2
**Kolliphor HS 15**	Millipore	Cat#: 42966-1KG
**1-methyl-2-pyrrolidinone**	Millipore	Cat#: 443778-500 mL
**D-α-tocopherol polyethylene glycol 1000 succinate**	Millipore	Cat#: 57668-5G
**Lipofectamin 2000 Transfection Reagent**	Thermo Fisher Scientific	Cat#: 11668019
**Hydrogen peroxide**	Thermo Fisher Scientific	Cat#: H325-500
**Triton-X**	Thermo Fisher Scientific	Cat#: BP151-500
**Dimethyl Sulfoxide (DMSO)**	Thermo Fisher Scientific	Cat#: BP231-1
**Polybrene**	Millipore	Cat#: TR-1003
**Bovine Serum Albumin (BSA)**	Sigma-Aldrich	Cat#: A9576
**H2DCFDA**	Sigma-Aldrich	Cat#: D6883
**Plasmids**		
**pCL-ECO**	Addgene	Cat#: 12371
**pBABE-MMEJ**		Ref. 38
**Restriction enzymes**		

**I-Sce*I***	Invitrogen	Cat#: ER1771
**Growth factors**		
**Recombinant Murine SCF**	Peprotech	Cat#: 250-03
**Recombinant Murine IL-3**	Peprotech	Cat#: 213-13
**Recombinant Murine IL-6**	Peprotech	Cat#: 216-16
**Recombinant Human IL-6**	Peprotech	Cat#: 200-06
**Recombinant Human SCF**	Peprotech	Cat#: 300-07
**Recombinant Human IL-3**	Peprotech	Cat#: 200-03
**Recombinant Human FLT3 ligand**	Peprotech	Cat#: 300-19
**Recombinant Human TPO**	Peprotech	Cat#: 300-18
**Inhibitors**		
**Disulfiram**	SelleckChem	Cat#: S1680
**N6022**	SelleckChem	Cat#: S7589
**ART558**	SelleckChem	Cat#: S9936
**RP-6885**	SelleckChem	Cat#: E1528
**Ruxolitinib**	SelleckChem	Cat#: S1378
**Quizartinib**	SelleckChem	Cat#: S1526
**L-BSO**	MedChem Express	Cat#: HY-106376A
**Mice**		
**Wild-type**	Jackson Laboratory	Cat#: JAX000664
Polq $^{-/-}$	Jackson Laboratory	Cat#: JAX006194
Adh5 $^{-/-}$	Jonathan Stamler	
Aldh2 $^{-/-}$	Lopa Mishra	
**Fox Chase SCID Beige Mouse**	Charles River	Cat#: 250
**Softwares**		
**Biorender**	Biorender	Biorender.com
**Prism GraphPad 10**	GraphPad	graphpad.com
**ImageJ**	NIH	imagej.net/ij/
**FlowJo 10.1**	FlowJo LLC	flowjo.com

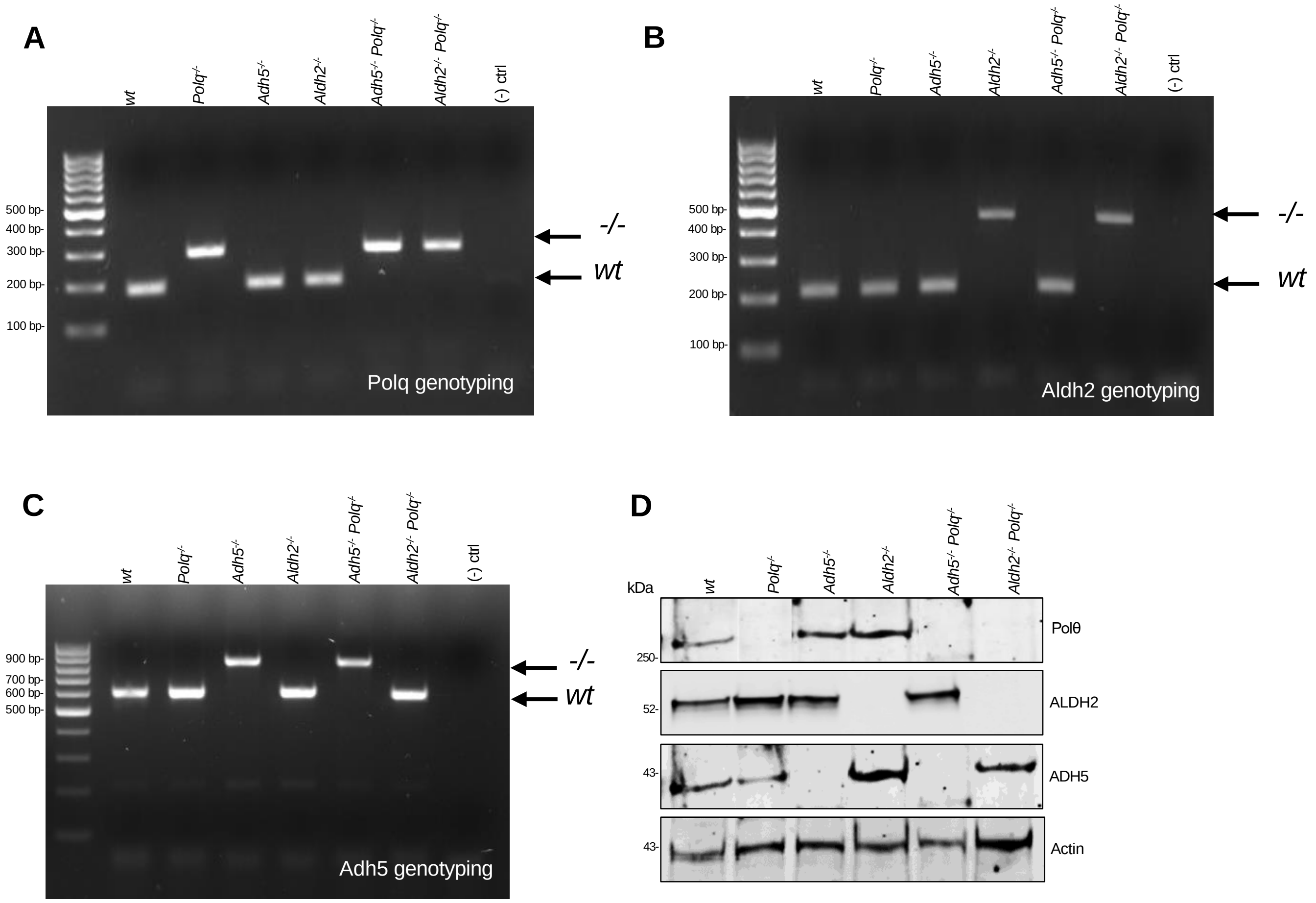

Supplemental Figure S1: Validation of genetic knockout mice. (A) PCR using *Polq* primers. Wild-type band is located at 190 bp while mutant band is located at 300 bp. **(B)** PCR using *Aldh2* primers. Wild-type band is at 212 bp while the mutant band is at 414 bp. **(C)** PCR using *Adh5* primers. Wild-type band is at 600 bp while the mutant band is at 900 bp. **(D)** Western blot analysis of Polθ (290 kDa), ADH5 (40 kDa), ALDH2 (56 kDa), and Actin (42kDa) detected in mouse HSPCs.

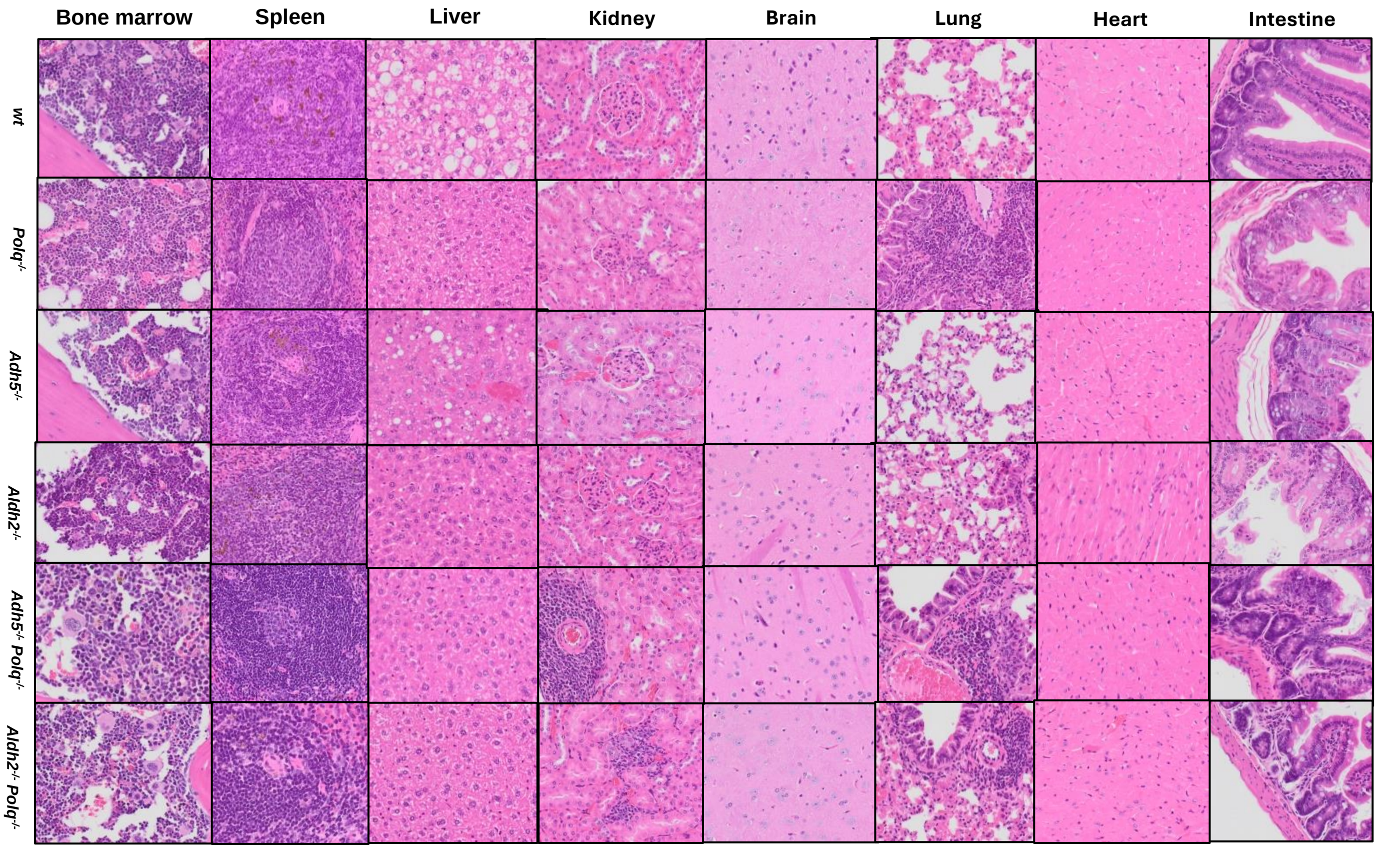

Supplemental Figure S2. No organ dysfunction from physiological levels of formaldehyde detected in aged (>18 months old) double knockout mice. Histopathology/H&E-stained tissue sections of the bone marrow, spleen, liver, kidney, brain, lung, heart, and bowel harvested from aged mice with the indicated genotypes (40x).

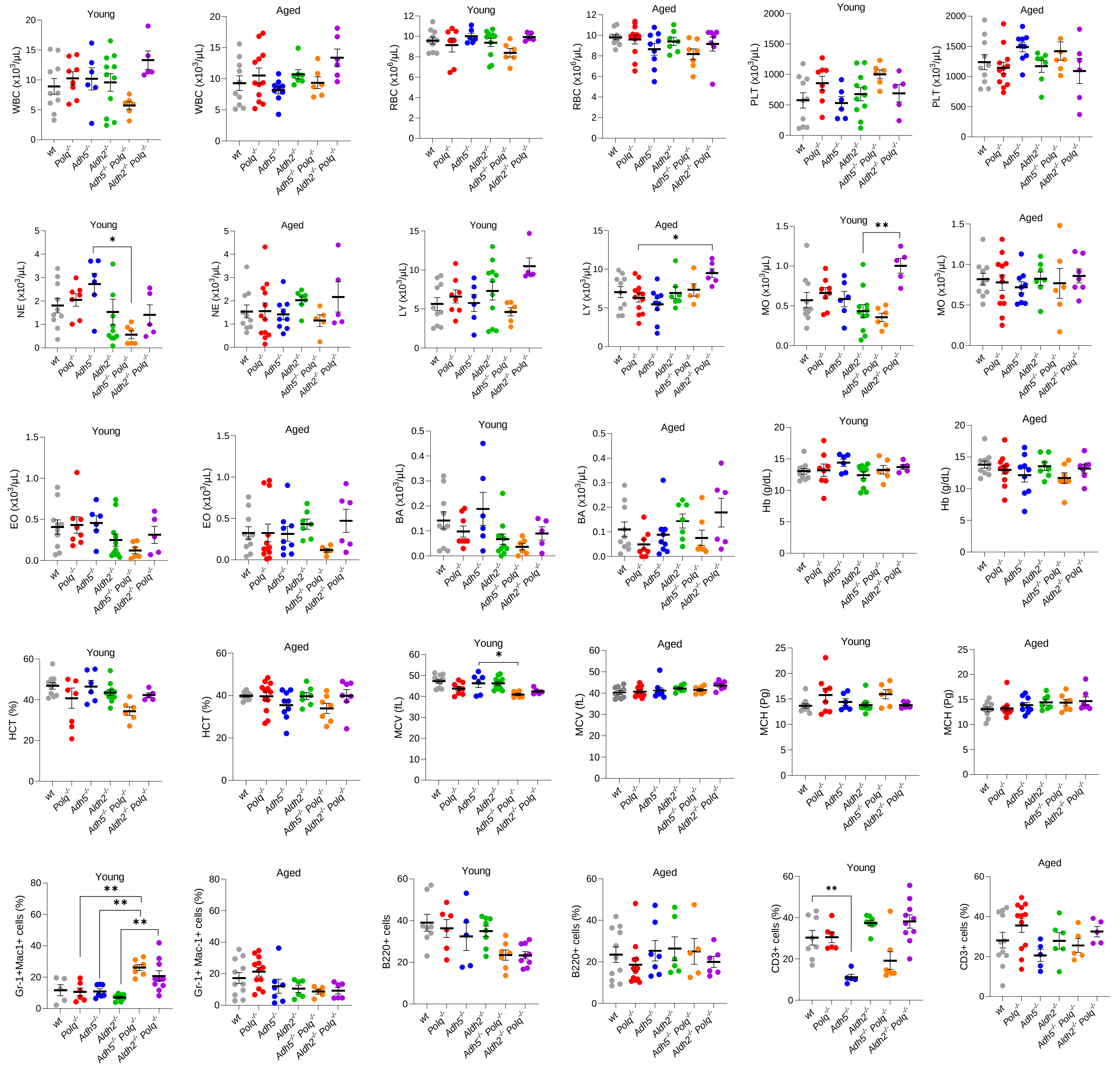

Supplemental Figure S3. Polθ and ADH5 or ALDH2 do not cooperate to protect young (3-4 months old) and aged (>18 months old) mice from physiological levels of formaldehyde. Peripheral blood parameters of the indicated genotypes of young and aged mice: white blood cells (WBCs), red blood cells (RBCs), platelets (PLT), neutrophils (NE), lymphocytes (LY), monocytes (MO), eosinophils (EO), basophils (BA), hemoglobin (Hb), hematocrit (HCT), mean corpuscular volume (MCV), mean corpuscular hemoglobin (MCH), Gr-1⁺Mac-1⁺ cells, B220⁺ cells and CD3⁺ cells. Each dot represents a result from an individual mouse.

Statistical analysis was performed using one-way ANOVA and Tukey's test for multiple comparisons where *p ≦ 0.05 and **p ≦ 0.01.

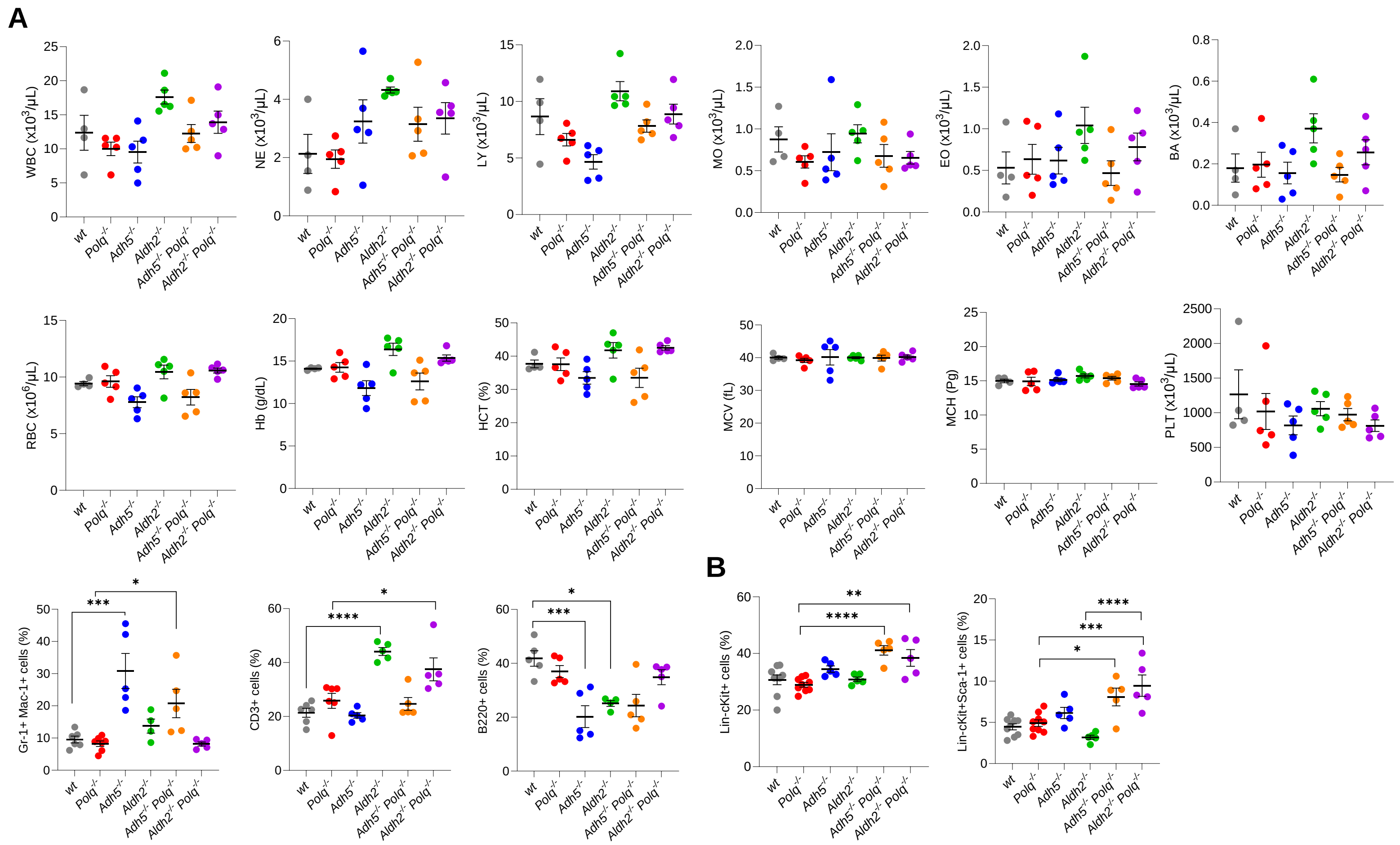

Supplemental Figure S4. *In vivo* methanol challenge did not significantly alter the basic peripheral blood or bone marrow parameters in *Adh5*$^{-/-}$;*Polq*$^{-/-}$ and *Aldh2*$^{-/-}$;*Polq*$^{-/-}$ mice. (A-B) Mean ± SEM of the indicated hematological parameters in peripheral blood **(A)** and bone marrow **(B)**.

Statistical analysis was performed using one-way ANOVA and Tukey's test for multiple comparisons where *p ≦ 0.05, **p ≦ 0.01, ***p ≦ 0.001 and ****p ≦ 0.0001.

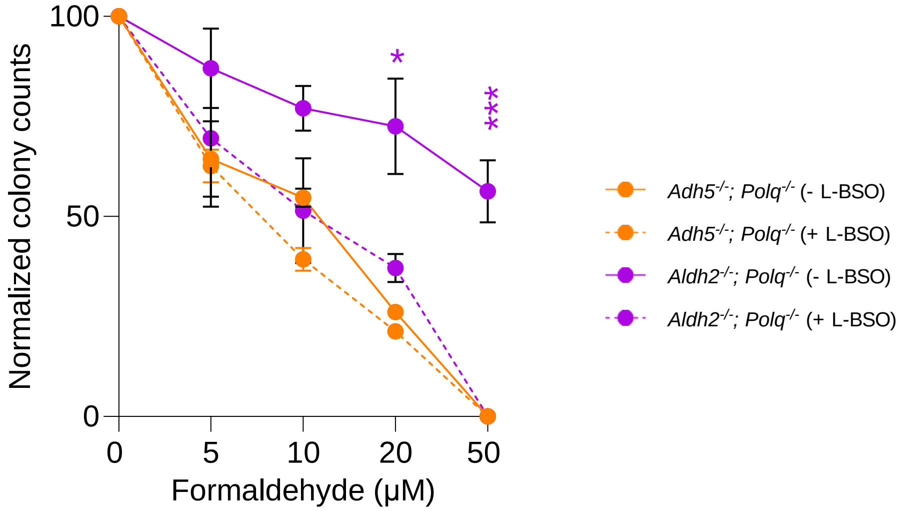

Supplemental Figure S5. GSH synthesis inhibitor (L-BSO) enhances synthetic lethality in *Aldh2$^{-/-}$;Polq$^{-/-}$* Lin-cKit+ BMCs. *Adh5$^{-/-}$;Polq$^{-/-}$* and *Aldh2$^{-/-}$;Polq$^{-/-}$* Lin-cKit+ BMCs were treated for 24 hours with the indicated concentrations of formaldehyde in the presence (+) and absence (-) of 50 µM L-BSO following the plating in methylcellulose. Colonies were counted after 7 days. Results show mean % of colonies ± SEM. Statistical significance was calculated using unpaired t-test to compare L-BSO treated and untreated counterparts; *p ≦ 0.05 and ***p ≦ 0.001.

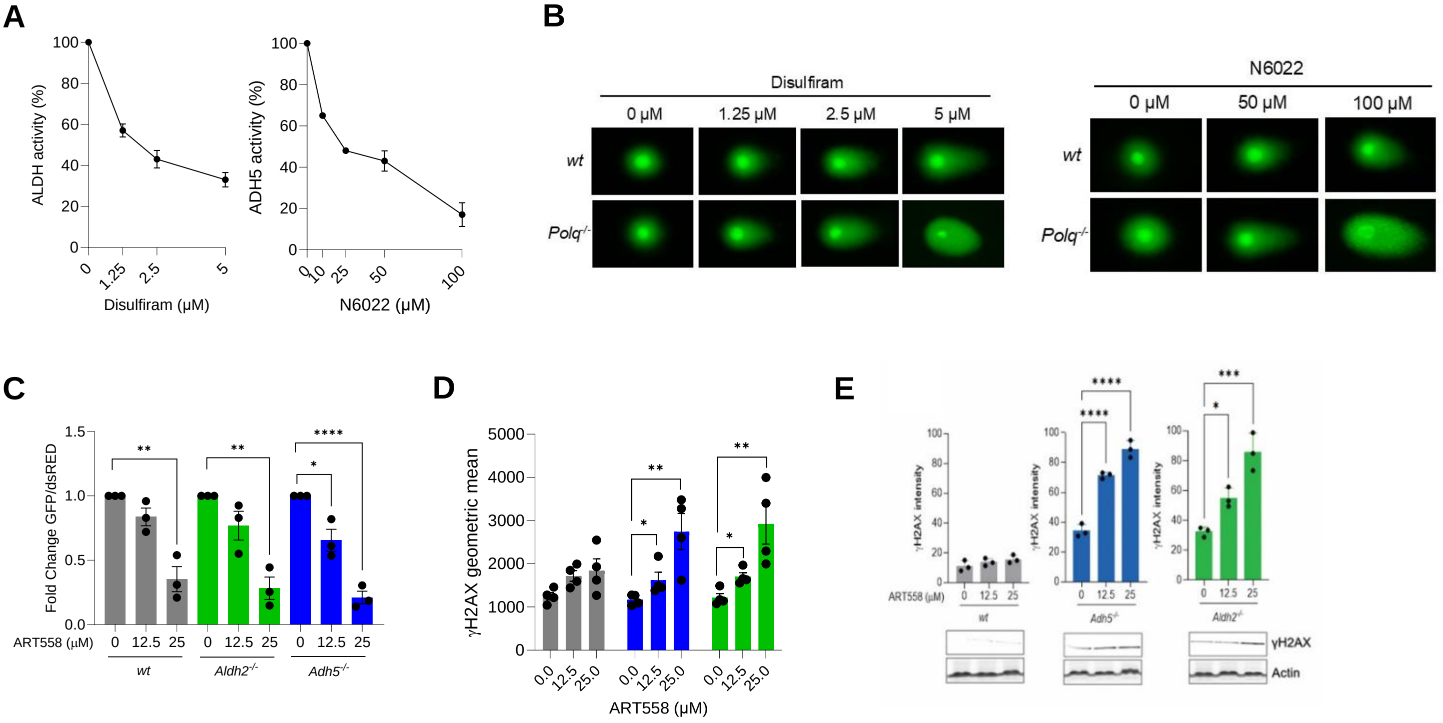

Supplementary Figure S6. Simultaneous inhibition of Polθ and ADH5 or ALDH2 is toxic for HSPCs. **(A-B)** Wild-type and *Polq*$^{-/-}$ Lin-cKit+ BMCs were treated with the indicated concentrations of disulfiram or N6022 for 48 hours. **(A)** ALDH and ADH5 activity in wild-type and *Polq*$^{-/-}$ Lin-cKit+ BMCs (n=3 mice/genotype) treated with disulfiram and N6022. Results represent % activity ± SEM when compared to untreated cells. **(B)** Representative comets are shown from neutral comet assay. **(C-E)** Wildtype, *Adh5*$^{-/-}$ and *Aldh2*$^{-/-}$ Lin-cKit+ BMCs were treated with the indicated concentrations of ART558 for 72 hours. **(C)** Mean ± SEM of TMEJ activity. **(D)** Mean ± SEM of γH2AX immunofluorescence determined by flow cytometry. **(E)** Representative western blots detecting γH2AX (lower panels). Quantification of γH2AX protein levels (upper panels).

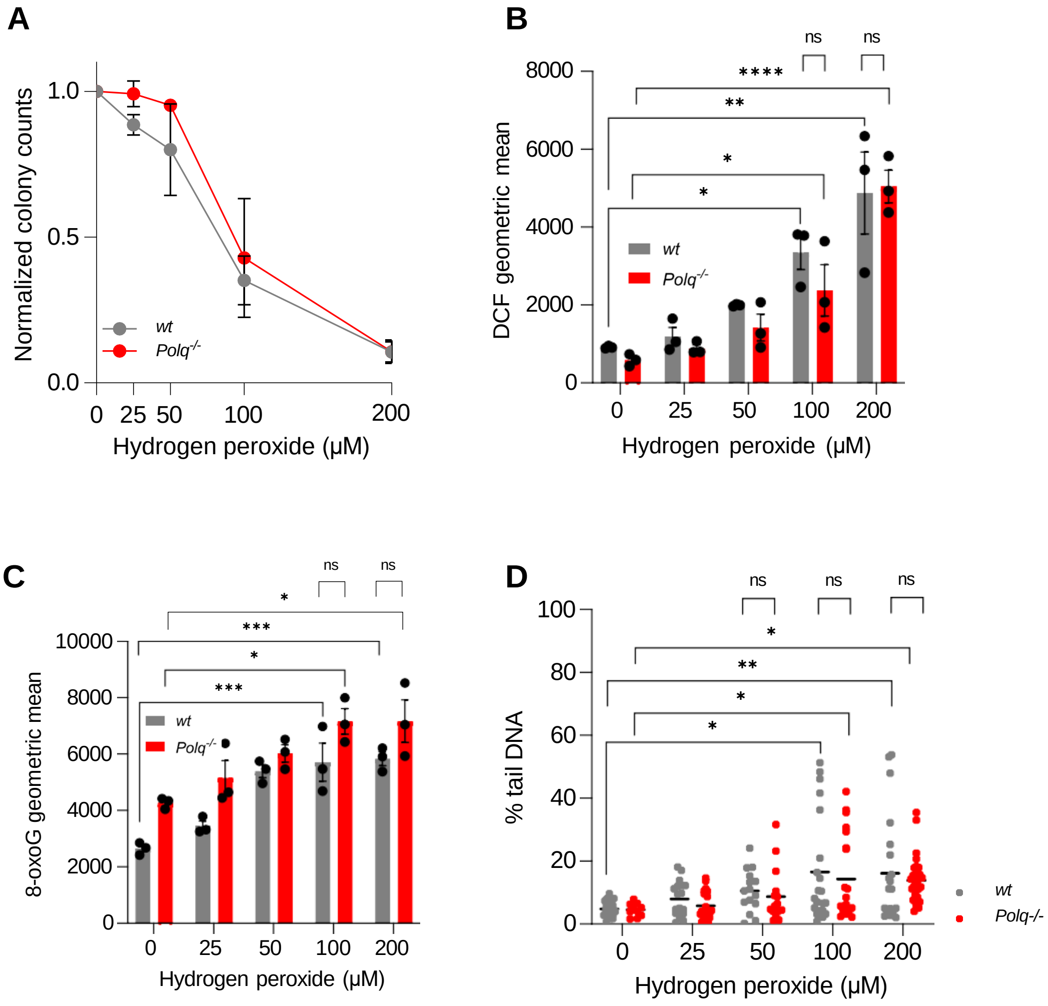

Supplemental Figure S7. *Polq$^{-/-}$ m*BMCs are not sensitive to oxidative DNA damage. Wild-type and *Polq$^{-/-}$* Lin-cKit+ mBMCs were treated with various concentrations of hydrogen peroxide for 4 hours. **(A)** Mean % colonies ± SEM in methylcellulose. **(B)** ROS was assessed by detecting mean DCF fluorescence ± SEM. **(C)** Mean 8-oxoG fluorescence ± SEM. **(D)** Mean % tail DNA ± SEM from neutral comet assay.

All results represent 3 independent experiments. Statistical analysis was performed using one-way ANOVA and Tukey's test for multiple comparisons when comparing different doses within each genotype where *p ≦ 0.05, **p ≦ 0.01, and ***p ≦ 0.001. Statistical analysis was also performed using unpaired t-tests when comparing *wt* vs. *Polq$^{-/-}$*.

A

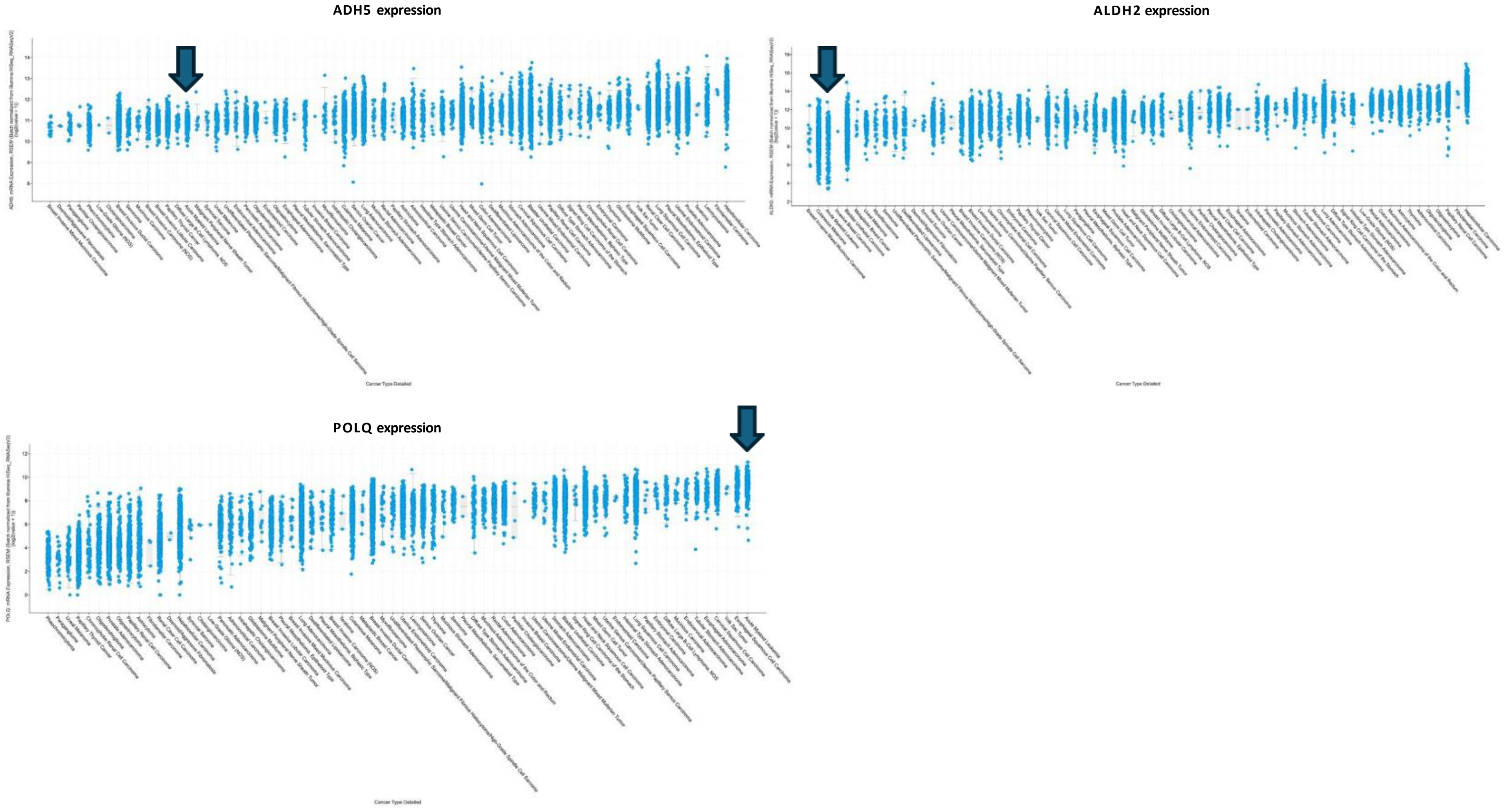

B

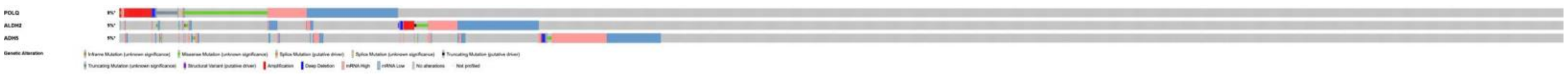

Supplemental Figure S8. Global analysis of the mRNA expression and genetic alterations of *ADH5, ALDH2* and *POLQ*. (A) The mRNA expression of *ADH5* (left), *ALDH2* (right) and *POLQ* (below) in various tumors in TCGA datasets; arrows indicate AML. **(B)** Co-occurrence of *ADH5, ALDH2,* and *POLQ* alterations in 10967 tumor samples from TCGA datasets.

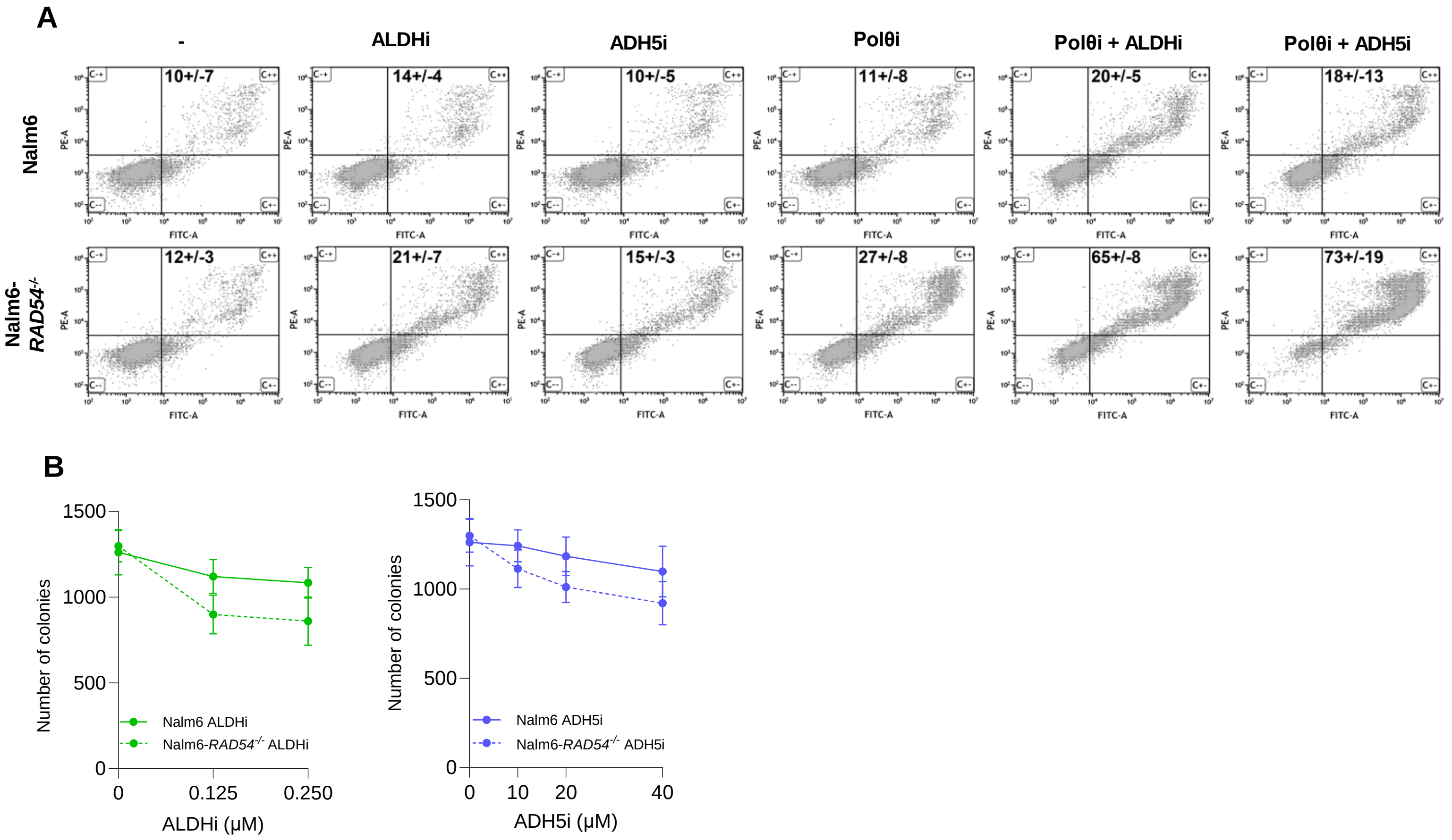

Supplemental Figure S9. Combination of Polθi +ALDHi and Polθi + ADH5i induced apoptosis in HR- deficient Nalm6-*RAD54*$^{-/-}$ cells. (A) Visual representation of Nalm6 and Nalm6-*RAD54*$^{-/-}$ cells treated with 12.5μM Polθi, 0.125μM ALDHi, 20 μM ADH5i, and indicated combinations for 48 hours followed by Annexin V/propidium iodide staining. Results represent mean % ± SEM of the double-stained cells. **(B)** Nalm6 and Nalm6-*RAD54*$^{-/-}$ cells treated with different concentrations of ALDHi (left) or ADH5i (right) for 48 hours followed by clonogenic assay.

(A) Statistical significance was calculated using one-way ANOVA and Tukey's test for multiple comparisons. Nalm6-*RAD54*$^{-/-}$ cells: Polθi vs. Polθi + ALDHi $p = 0.0015$, ALDHi vs. Polθi + ALDHi $p = 0.00063$, Polθi vs. POLθi + ADH5i $p = 0.0041$, and ADH5i vs. Polθi + ADH5i $p = 0.0035$.
